# A narrative review: predicting liver transplant graft survival using artificial intelligence modeling

**DOI:** 10.3389/frtra.2024.1378378

**Published:** 2024-05-13

**Authors:** Aiste Gulla, Ieva Jakiunaite, Ivona Juchneviciute, Gintautas Dzemyda

**Affiliations:** ^1^ Faculty of Medicine, Institute of Clinical Medicine, Vilnius University, Vilnius, Lithuania; ^2^ Faculty of Medicine, Vilnius University, Vilnius, Lithuania; ^3^ Faculty of Mathematics and Informatics, Institute of Data Science and Digital Technologies, Vilnius University, Vilnius, Lithuania

**Keywords:** liver transplantation, short-term liver graft survival, long-term liver graft survival, artificial intelligence algorithms, liver transplantation characteristics

## Abstract

Liver transplantation is the only treatment for patients with liver failure. As demand for liver transplantation grows, it remains a challenge to predict the short- and long-term survival of the liver graft. Recently, artificial intelligence models have been used to evaluate the short- and long-term survival of the liver transplant. To make the models more accurate, suitable liver transplantation characteristics must be used as input to train them. In this narrative review, we reviewed studies concerning liver transplantations published in the PubMed, Web of Science, and Cochrane databases between 2017 and 2022. We picked out 17 studies using our selection criteria and analyzed them, evaluating which medical characteristics were used as input for creation of artificial intelligence models. In eight studies, models estimating only short-term liver graft survival were created, while in five of the studies, models for the prediction of only long-term liver graft survival were built. In four of the studies, artificial intelligence algorithms evaluating both the short- and long-term liver graft survival were created. Medical characteristics that were used as input in reviewed studies and had the biggest impact on the accuracy of the model were the recipient's age, recipient's body mass index, creatinine levels in the recipient's serum, recipient's international normalized ratio, diabetes mellitus, and recipient's model of end-stage liver disease score. To conclude, in order to define important liver transplantation characteristics that could be used as an input for artificial intelligence algorithms when predicting liver graft survival, more models need to be created and analyzed, in order to fully support the results of this review.

## 1 Introduction

Liver transplantation is one of the most complex fields of medicine, demanding precision not only during the transplant surgery itself or caring for the patient in the perioperative period, but also in selecting suitable donors and recipients, as well as estimating the survival of a liver graft (1). Many patients die while waiting for liver transplants since the demand for donated livers far exceeds the supply. Despite this reality, the number of transplants does not significantly increase over time, although more extended criteria organs (such as organs after circulatory death of the patient) are used for liver transplantation (2–4). Because donor organs are a scarce resource, it is becoming increasingly important to increase liver graft utilization and, at the same time, to ensure that the best possible outcomes can be achieved (1). Until now, various scores [balance of risk (BAR), model of end-stage liver disease (MELD), model of end-stage liver disease (including serum sodium concentration) (MELD-Na), Child–Pugh, survival outcome following liver transplantation (SOFT), and others] have been used worldwide to address this issue (2, 5–7). The number of points scored in these scales determines whether a patient needs a liver transplant and how quickly it should be performed, and assesses the patient's mortality rate after the transplant (8). However, the assessments made using these scales do not always accurately reflect the urgency and necessity of transplantation or the patient's post-transplant outcome. For example, the MELD score system is now widely used to prioritize the patients who are waiting for liver transplantation. However, some of its results might not be completely reliable (9), as the association between the pre-transplantation MELD score and post-transplant survival represents a low level of evidence (6). Other scores, such as BAR and SOFT, are also used to facilitate surgical decision making (8). However, survival prognosis for the recipient is believed to be an extremely complex relationship that is non-linear in nature (10). It is observed that optimal graft allocation as well as short- and long-term graft survival are dependent on many different characteristics, such as the recipient's or donor's demographic data, laboratory findings, chronic diseases, and other variables. Therefore, more reliable methods are being sought to evaluate the survival of liver graft recipients (11).

Artificial intelligence algorithms, used to calculate the survival of the patient after liver transplantation, would be more efficient than existing scores (2). Machine learning (ML) algorithms can be used to predict the outcome of a new observation, based on a training set containing previous observations where the outcome is known (12). Using pre-transplant characteristics of donors and recipients, machine learning models can predict short- and long-term patient survival after transplant with higher accuracy than advanced biostatistical models, predominantly due to the ability to integrate a larger number of variables and data types (11). However, it should be noted that machine learning techniques need a precise set of operating conditions to perform well. The input data must be adequately processed and input variables should be chosen carefully in order not to downgrade the algorithm's performance (13). As the outcome after liver transplantation depends upon a complex interaction between donor, recipient, and process factors, choosing the input variables for the machine learning algorithms tends to be one of the main issues while applying neural networks (NN) in liver transplantation (12). The estimations made in clinical practice using a neural network algorithm might also not be accurate when some of the variables are missing, for example, when the donor information cannot be used as input as it is not always available in advance (14). Another challenge is choosing which particular algorithm should be used in order to estimate the survival of the graft. Currently, random forest (RF), support vector machine (SVM), and artificial neural networks (ANNs) are mostly used in medical decision making (1). Artificial neural networks imitate human thinking as they gather their knowledge by detecting the patterns and relationships in data and learn (or are trained) through experience, not from programming (15). The random forest, on the other hand, uses randomization to create a large number of decision trees (DTs). Then, the algorithm chooses which combination of the variables differs the most from the control group (16). The support vector machine classifies objects as points in an interdimensional space and draws multiple planes, which could separate objects of two separate groups in a most effective way (17). Deciding which method of machine learning works best on the data depends on many factors; therefore, most of the time more than one algorithm is applied to find the model with best accuracy.

In this work, we analyzed the current literature of neural network applications in evaluating short- and long-term survival after liver transplantations. We have picked out the most important and precise characteristics used in the neural networks as inputs, as well as evaluated the drawbacks their usage may have when predicting the short- and long-term survival of the liver graft.

## 2 Methods

We defined short-term liver graft survival as less than 6 months after transplantation and long-term liver survival as more than 6 months after transplantation. We then searched the PubMed, Cochrane, and Web of Science databases to find articles concerning artificial intelligence models used to evaluate the survival of liver grafts. The keywords we used were: “Liver Transplantation”, “Artificial Intelligence”, “Neural Network”, “Machine Learning”, “Deep Learning”, and “Statistical Model”. After our initial search, we had 976 results. We then applied inclusion and exclusion criteria to the articles. The inclusion criteria were as follows: studies published between 2017 and 2022, studies concerning short- and long-term graft survival after liver transplantation, studies concerning adult liver transplantation, and studies in English.

The exclusion criteria were as follows: case reports; systematic reviews, literature reviews, or meta-analyses; abstract-only publications; studies concerning pediatric liver transplantation; studies concerning multi-organ transplantation; studies concerning liver transplantation candidate survival and mortality; and studies with algorithms created to evaluate radiology images or biopsies.

## 3 Results

### 3.1 Literature review

We selected the literature for our narrative review by searching the PubMed, Cochrane, and Web of Science databases, and applying exclusion and inclusion criteria to the selected studies. After our initial search of the aforementioned databases, we had 976 results. We then applied the inclusion and exclusion criteria to the articles and 958 studies were excluded. Only 18 articles remained. One of the articles was unavailable due to fees. Therefore, we finally had 17 articles for our literature analysis.

**Figure 1:**
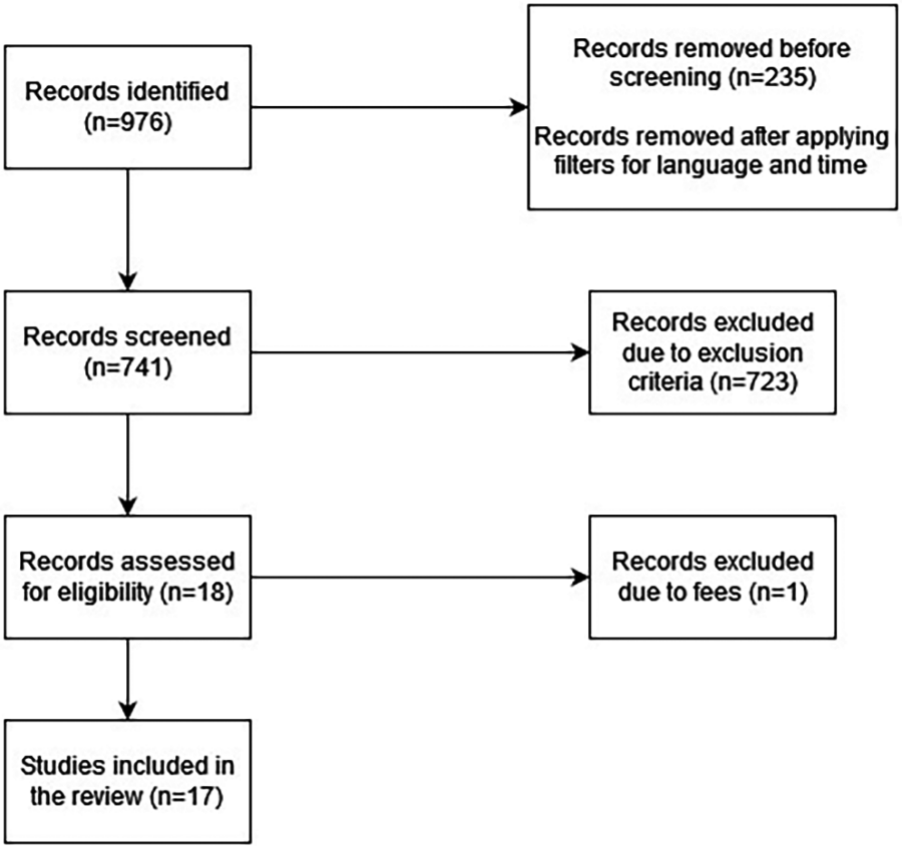
Identification of studies via databases.

The process of identification of studies is represented in Figure 1.

Most of the studies (six studies) were carried out in the USA (18–23). Six of the studies were conducted in Asia (14, 24–28) and there were three European studies (2, 13, 29), one Australian study (12), and one Canadian study (30).

### 3.2 Artificial intelligence models

The included studies handled the problem of evaluating short- and/or long-term liver graft survival and choosing types of artificial intelligence models that could be the most appropriate for survival prediction. In eight studies, models estimating only short-term (up to 6 months) liver graft survival were created (12, 14, 21, 22, 26–29), while in five of the included studies, models for the prediction of only long-term liver graft survival were built (11, 13, 26–28). In four studies, models predicting both short- and long-term survival were created (2, 18, 23, 24). Eight of the studies used large datasets (including more than 10,000 patients) (2, 11, 13, 19–23), whereas four of the studies included a medium number of patients (1,000–10,000 patients) (14, 18, 19, 28). Small datasets (including less than 1,000 patients) were used in six studies (12, 24–27, 29).

Most of the studies (12 studies) used more than one type of artificial intelligence algorithm (2, 11–14, 19–21, 23–28). Brain-inspired machine learning algorithms were used in 10 studies: deep neural networks (DNNs) were used in three studies (2, 22, 23), artificial neural networks were applied in five studies (12–14, 20, 27), and a multilayer perception neural network was used in two studies (25, 26). Popular machine learning algorithms for classification, such as logistic regression (LR) (used in eight studies) (2, 11, 12, 14, 20, 21, 26, 27), random forest (used in seven studies) (2, 11, 12, 24, 26–28), support vector machine (used in four studies) (11, 24–26), and decision tree (used in three studies) (2, 24, 27) were also widely used in the aforementioned studies. Cox models were applied in five studies (13, 19, 24–26). Yu et al. (24) and Yang et al. (26) not only applied machine learning models but also compared their efficacy with the conventionally used MELD score.

Most of the studies use large databases and more than one type of artificial intelligence algorithm when creating artificial intelligence models for the prediction of liver graft survival.

### 3.3 Study characteristics

To create an accurate artificial intelligence model, certain variables must be picked out to train the algorithm. Here, we discuss the variables that were deemed most important in our selected studies – having most impact for the accuracy of the model.

In most of the studies, up to 50 variables were used as an input to train artificial intelligence models (11, 14, 18, 19, 21, 24, 25, 27–29); however, there were four studies that used more variables. Two studies used up to 100 variables (13, 20), in two models up to 200 variables were used (20, 23), and there were two studies that used more than 200 variables as an input (2, 22). In the study by Lau et al. (12), more than 200 variables were used initially; however, after selection, only 15 variables were included in the most accurate model.

The most important characteristics were selected in only 16 studies, as they were not mentioned in the study by Ershoff et al. (22). Among the most important variables, the demographic data of the recipient and donor played an important role. The recipient’s age was selected as an important variable in 12 of the studies (2, 11, 13, 14, 18–21, 23, 24, 27, 29), whereas the donor's age was a significant variable in four studies (2, 13, 20, 23). The recipient's sex was significant in two studies (11, 29). Anthropometric data, such as the weight and BMI of the recipient, were also among the important variables. The recipient's BMI was a significant variable in six studies (19, 21, 23, 24, 27, 29) and the recipient's weight was mentioned as a relevant characteristic in two studies (24, 29). Laboratory findings were indicated as significant in almost all studies. Creatinine levels in the recipient's serum were an important variable in five studies (2, 14, 19, 26, 29), and bilirubin levels in the recipient's serum were mentioned as a relevant characteristic in two studies (14, 19). The recipient's international normalized ratio (INR) was also a significant variable in four studies (24, 26, 27, 29) and albumin was deemed to be relevant in models of two studies (18, 24). The impact of hyperlactatemia on liver graft survival was analyzed in detail in the study by Cheong et al. (28). The comorbidities of the recipient were also included in the list of relevant characteristics of the liver transplantation: the recipient having diabetes mellitus (DM) was mentioned among the important characteristics in five studies (13, 18, 20, 21, 25) and its importance was thoroughly analyzed in the study by Bhat et al. (30), in which they researched the impact of new-onset and pre-existing diabetes mellitus on liver graft survival. Dialysis before transplantation was also mentioned as relevant in two studies (21, 29). Among the scores used in liver transplantation, only the MELD score was selected as an important characteristic in five of the studies (20, 21, 23, 25, 28). Cold ischemic time was a significant variable in three studies (2, 13, 24), whereas other characteristics, such as donor intensive care unit (ICU) stay (13, 24) and length of hospital stay (2, 18), were mentioned as important in only two studies.

According to the selected studies, the most important variables were the recipient's age, recipient's BMI, creatinine levels in the recipient's serum, recipient's INR, diabetes mellitus, cold ischemic time, and recipient's MELD score. Using 50 characteristics or less as a training input for the artificial intelligence models seemed to be the most preferred decision in our selected studies.

### 3.4 Metrics of survival prediction

After creating the artificial intelligence algorithm, the accuracy of the model is measured to estimate whether the model could be applicable in everyday clinical decisions (2).

The accuracy of the models in 12 of our selected studies was compared using the area under the curve (AUC). A model with an AUC higher than 0.9 was created in only four studies (21, 25, 26, 29). Other models in six studies reached an AUC in the range of 0.7–0.9 (2, 12, 20, 22, 24, 27). An AUC below 0.7 was reached in only two studies (2, 23). The AUC of the models was not measured in six studies (11, 13, 14, 18, 23, 28). In the study by Kong et al. (14), the model C-statistic was measured and reached the highest result of 0.668 in the original model. In the study by Bhat et al. (30), the squared error of the model was calculated and achieved 0.1059 in predicting diabetes mellitus 1 year after transplantation. In the study by Andres et al. (18), only the calibration of the model was measured and the *p*-value of 0.278 suggested good calibration. In two studies, the C-index was used to estimate the accuracy of the models. In the study by Kantidakis et al. (13), the RF model achieved a C-index of 0.622. In the study by Farzindar and Kashi (23), the C-index results of 0.82 on the Scientific Registry of Transplant Patients (SRTR) database and 0.57 on the united network of organ sharing (UNOS) database were achieved. In the study by Cheong et al. (28), the accuracy of the model was not measured.

In summary, most of our selected studies managed to create models with sufficient accuracy and good calibration.

The summary of each study can be seen in Tables 1–3.

**Table 1:** Short-term survival.

Reference	Dataset	Number of variables	Top predictive characteristics	Models used	Best model accuracy
Liu et al. (27)	480 recipients	17 variables	BMI, age, Na, lymphocyte, INR, WBC, platelets, Mg	RF, XG-Boost, DT, LR	AUC:0.771, specificity: 0.815
Ershoff et al. (22)	57,544 recipients	202 variables	—	DNN	AUC: 0.703
Molinari et al. (21)	30,458 recipients	13 variables	Recipient age, MELD score, BMI, diabetes, and dialysis before transplantation	ANN, LR, CTA	AUC: 0.952 (90-day mortality)
Börner et al. (29)	529 recipients	48 variables	Age, sex, weight, BMI, dialysis, serum potassium, ALT, INR, creatinine levels	NN	Accuracy: 94.3%, AUC: 0.940
Kong et al. (14)	1,495 recipients	33 variables	Creatinine, age, total bilirubin, albumin	ANN, LR	Original model C-statistic: 0.668; simplified model C-statistic: 0.647.
Yang et al. (26)	132 recipients	—	Creatinine, INR	CR, SVM, LR, RF, MLP	RF model—highest accuracy: AUC: 0.940
Cheong et al. (28)	2,002 recipients	10 variables	Hyperlactatemia, MELD score, circulatory failure, hemoglobin, respiratory failure	RSF, Kaplan–Meier survival curve analysis	Kaplan–Meier analysis showed clear separation of survival curve of 90-day mortality between patients with low and patients with high lactatemia (>4 mmol/L)
Lau et al. (12)	180 recipients	276 variables; after selection: 15 variables	Recipient disease, donor serum albumin level, donor cause of death, donation after brain or cardiac death	RF, ANN, LR	RF with the top 15 donor and recipient characteristics achieved AUC-ROC of 0.818

CTA, classification tree analysis; MLP, multilayer perceptron neural network; RSF, random survival forest.

**Table 2:** Long-term survival.

Reference	Dataset	Number of variables	Top predictive variables	Models used	Best model accuracy
Nitski et al. (20)	42,146 recipients and 3,269 recipients	190 and 63 variables	Donor age, recipient age, MELD score	Deep learning models and baseline LR model	First model AUC: 0,804 (1-year survival); 0,733 (5-year survival). Second model: AUC: 0.807 (1-year survival); 0,722 (5-year survival)
Kantidakis et al. (13)	62,294 recipients	97 variables	Re-transplantation, donor age and type, total cold ischemic time, diabetes, black race, life support, recipient age, incidental tumor, HCV, ICU	Cox models (all variables, backward selection and LASSO), RSF and two partial logistic ANN	Best model: random survival forest with C-index of 0.622
Yasodhara et al. (19)	18,058 and 1,290 recipients	26 variables	Bilirubin, high creatinine, BMI, recipient's age	Cox proportional hazards and gradient boosting survival models	AUC 0.6 and 0.7
Kazemi et al. (25)	902 recipients	26 variables	Graft failure, *Aspergillus* infection, acute renal failure, vascular complications, graft failure, diagnosis interval, previous diabetes mellitus, MELD	CR, C5.0 DT, SVM, BN, KNN, MLP	AUC: 0.90; sensitivity 0.81
Bhat et al. (30)	61,677 recipients	17 variables	Increasing age, male sex, obesity, use of sirolimus, tacrolimus, diabetes mellitus	High-performance RF, high-performance NN, gradient boosting, high-performance SVM, LR	High-performance random forest (0.1059 average squared error in predicting diabetes mellitus first year after liver transplantation)

BN, Bayesian network; MLP, multilayer perceptron neural network; KNN, K-nearest neighbors; RSF, random survival forest; ICU, intensive care unit; HCV, hepatitis C virus.

**Table 3:** Short- and long-term survival.

Reference	Dataset	Number of variables	Top predictive variables	Models used	Best model accuracy
Zhang et al. (2)	41,455 patients	217 variables	Age, donor age, serum creatinine, hospitalization, cold ischemia time	LogitBoost, LR, DT, RF, DNN, AdaBoost, extreme gradient boost	XG-Boost: AUC in all of 3-month survival: 0.717, 1-year survival: 0.681 3-year survival: 0.662, 5-year survival: 0.660, 10-year survival: 0.674
Yu et al. (24)	785 recipients	46 variables	Cold ischemic time, donor ICU stay, recipient weight, BMI, age, albumin, INR	RF, ANN, DT, naive Bayes, CR, SVM, MELD score, donor MELD score, BAR score	Random forest AUC values: (1-month = 0.80; 3-month = 0.85; 12-month = 0.81) for predicting survival
Andres et al. (18)	2,769 recipients	17 variables	Recipient age, hospitalization, diabetes, albumin	PSSP model	The *p*-value (Hosmer–Lemeshow) is 0.278, suggesting good calibration
Farzindar and Kashi (23)	59,115 recipients and 87,334 recipients	Around 150 variables	MELD score, recipient BMI, donor and recipient age	DNN, seep survival mode	C-index results of 0.82 and 0.57 on the SRTR and UNOS datasets, respectively

PSSP, patient specific survival prediction; ICU, intensive care unit; UNOS, united network of organ sharing.

## 4 Discussion

Today, transplant physicians are faced with the task of discussing the risk of postoperative death with potential transplant recipients. As this task is often challenging, simple scoring systems based on mental calculations are very useful in clinical practice (14). To help the physicians in their practice daily, MELD, BAR, Child–Pugh, SOFT, and other scores were created; however, to estimate the survival of a liver graft more accurately, artificial intelligence models can be applied (1). In order to create a highly specific and accurate model, the input characteristics used to train the models must be chosen carefully.

### 4.1 Recipient's age

One of the most important factors that reduce the survival of a liver graft is the recipient's age at the time of transplant (19). Age and mortality do not exhibit a linear relationship. However, when the patient's age is above 65 years, the mortality rate increases sharply (14). Although the age limits for liver transplantation are now widened, older age has an adverse effect on the survival of a liver graft (13). Older patients have a higher risk of cardiovascular mortality (19). Moreover, according to the study by Su et al. (31) on aging of liver transplant registrants and recipients, older patients are usually more prone to diabetes, hepatocellular carcinoma, non-alcoholic liver disease, and other comorbidities. Therefore, the 5-year survival probability after transplantation is higher in the 18–49-year group than in the >70-year group (78% in the first group and 62% in the second group). The recipient's increasing age also significantly increases the risk of new-onset diabetes after liver transplantation. Each year increase in the recipient’s age at the time of the transplant increases the odds of new-onset diabetes by 0.1%. This may affect the long-term survival after the transplant (11). The recipient's age also defines which risk factors can influence acute graft rejection or a higher risk of death after transplantation. It is indicated that the mortality risk for the older subgroups is more influenced by chronic diseases and geriatric conditions, which are variables that cannot be modified. For younger patients, features such as cold ischemic time, donor age, serum albumin, recipient weight, and BMI are the most important (2).

### 4.2 Recipient's BMI

BMI is generally used to evaluate the obesity of a patient. Preoperative visceral adiposity, as well as low muscularity, is closely involved with post-transplant mortality (32). Obesity is a risk factor for various health disorders, including type 2 diabetes mellitus, hypertension, cardiovascular disease, and non-alcoholic steatohepatitis (33). Obese patients are also more prone to comorbidities, such as gallstones and colon cancer (34), which may affect the patient's outcome and mortality after the liver transplantation. According to the study by Naoko et al. (35), patients with sarcopenic obesity had lower survival rates after liver transplantation than non-obese patients. Even if obese patients might have acceptable survival after transplantation, their body habitus makes them particularly susceptible to obesity-related complications and recurrence of non-alcoholic steatohepatitis (36).

Although the recipient's BMI seems to be an important characteristic of liver transplantation when estimating graft survival, it must be considered that BMI is an indirect measurement of adipose tissue and it cannot account for differences in fat distribution. This way, BMI is usually overestimated due to massive ascites and systemic edema in patients with end-stage liver disease who require liver transplantation (32). Therefore, it might be misleading to rely on the recipient's BMI alone to analyze his or her body constitution. To that matter, computed tomography imaging is used to evaluate the patient's body composition more accurately and distinguish between areas of visceral and subcutaneous adipose tissue (37).

### 4.3 Creatinine levels in patient's serum

Kidney function in patients with liver cirrhosis waiting for liver transplantation is dynamic. Yet, the ability to identify which patients will have the greatest variation of creatinine levels and understanding of the impact of this variation are limited. However, it can be seen that all fluctuations in serum creatinine levels are associated with worse pre- and post-liver transplantation outcomes, because it might indicate that the patient is at risk of experiencing acute kidney injury (38). In the study by Nacif et al. (39), higher creatinine levels in patients with hepatitis C virus was one of the predictors of mortality and late acute rejection in liver transplantation. Another study by Asrani et al. (40) indicated that a set of recipient factors, among which was higher (>1.5 mg/dl) creatinine levels, can help identify patients who may not do well after a transplant. As an overwhelming majority of liver transplantation recipients develop chronic kidney disease (41), which might be due to calcineurin inhibitor-toxicity, perioperative acute kidney injury, diabetes mellitus, hypertension, and chronic hepatitis C infection (42), it is crucial to assess the kidney function of the liver graft recipient as it might have a huge impact on the long-term survival of the patient.

### 4.4 Recipient's INR

Chronic liver disease, particularly in the advanced or decompensated stages, has historically been regarded as an example of an acquired bleeding diathesis primarily based on abnormalities in basic conventional laboratory tests of coagulation, such as prothrombin time, activated partial thromboplastin time, and INR (43). The higher the INR value, the worse the prognosis (27); INR is a distinct prognostic factor of poor short-term survival (26). In a study by Yu et al. (24), INR was a significant characteristic predicting a recipient's survival 3 months after a transplant using the random forest model, although it was not statistically significant in the Cox model. Another study by Okamura et al. (44) showed that total bilirubin of 10 mg/dl or greater and/or prothrombin time/INR of 1.6 or greater on postoperative day 7 predicted early graft loss after living donor liver transplantation, and their coexistence worsened patient outcomes.

However, it must be noted, that prothrombin time and INR are no longer accepted as means of determining thrombotic or bleeding risk in patients with cirrhosis (45), as these tests only measure the levels of procoagulant proteins and fail to account for the concurrent alterations in anticoagulant proteins or platelets that are known to occur in those patients (46). Clinicians should avoid making medical decisions based on these values alone without properly assessing the other components of the system (45).

### 4.5 Diabetes mellitus

Liver transplantation differs from other solid organ transplants, because diabetes mellitus is frequently observed before surgery in susceptible individuals, possibly favored by certain etiological agents of liver disease (47), which lead to so called “hepatogenous diabetes mellitus” (48). Diabetes mellitus is usually not included in widely used prognostic tools such as Child–Pugh and MELD (49); however, diabetes is an independent factor for poor prognosis in patients with cirrhosis as it is associated with the occurrence of major complications of cirrhosis, including ascites and renal dysfunction, hepatic encephalopathy, and bacterial infections as well as hepatocellular carcinoma (50). According to Gitto et al. (51), pre-transplant diabetes can predict the cardiovascular mortality of liver transplant patients, as it is the main risk factor for a post-liver transplantation atherosclerotic vascular event, which, together with diabetes mellitus, is a strong, long-term predictor of cardiovascular mortality. Thus, patients with pre-liver transplantation diabetes should obtain a personalized follow-up for the prevention or early diagnosis of atherosclerotic vascular events. Furthermore, new-onset diabetes after liver transplantation adversely affects the long-term survival of the liver graft in a manner similar to pre-existing diabetes. This indicates the need to be vigilant and implement close follow-up regarding glycemic control in patients with new-onset diabetes after transplantation to maximize their survival (30).

### 4.6 MELD score

The MELD score is calculated using bilirubin, INR, and creatinine and could be considered as a combination of the three features (27). It can be one of the features usually used when creating a model for predicting the survival of a liver graft (20). In a study by Molinari et al. (21), MELD, among other factors, such as recipient’s age, BMI, dialysis, and diabetes, was one of the strongest independent predictors for 90-day mortality. Moreover, the MELD score is used to predict hyperlactatemia after liver transplant as it is related to hepatic dysfunction leading to reduced metabolism of lactate (28).

Although the MELD score is widely used in organ allocation practice, it fails to accurately predict the survival of a graft, as it only considers a few factors of the recipient. The SOFT score and BAR score, on the other hand, consider the factors of both the recipient and the donor and therefore show better results at predicting the recipient's mortality after transplantation (2).

### 4.7 Artificial intelligence models

In our analyzed studies, the artificial intelligence methods that were mainly used were random forest and logistic regression. Both methods can be used to select the most important features for the model (27) as well as for the prediction of liver graft survival (1). Among other artificial intelligence models that are usually mentioned in the literature as applicable for liver graft survival evaluation, support vector machine, artificial neural networks, and random forest are mentioned (1, 12, 24, 27). As there are so many different algorithms that can be applied, it is hard to argue which model is the best for liver graft survival prediction.

### 4.8 Limitations of the study

In this study, due to strict study selection criteria, we only scrutinized 17 studies; therefore, our results and conclusions could be limited due to the number of studies examined. Moreover, analyzing studies that are related to the use of artificial intelligence not only in estimating the survival of the liver graft, but also in donor-recipient matching, predicting the risk of hepatocellular carcinoma recurrence after liver transplantation, and other liver transplantation fields could give us more detailed findings about transplantation characteristics that have the biggest impact on the accuracy of the artificial intelligence model.

## 5 Conclusions

Machine learning and artificial intelligence offer new working styles for managing liver transplantation, impacting both early graft and patient survival. These technologies hold the potential to enhance predictive accuracy and influence surgical decisions. In addition, they can identify critical intervals of donor and recipient factors, parameters, and features, thereby potentially improving surgical outcomes, reducing complications, and optimizing pre- and postoperative care.

In this narrative review, we analyzed 17 studies to find which liver transplantation factors have the biggest influence on the accuracy of artificial intelligence models when predicting graft survival. We can conclude that recipient's age, recipient's BMI, creatinine levels in recipient's serum, recipient's INR, diabetes mellitus, and recipient's MELD score tend to be important variables in most artificial intelligence models when estimating the short- and long-term survival of liver recipients. The most popular artificial intelligence models for the prediction of liver graft survival among our selected studies were random forest and logistic regression.

Directions for further research also emerge. First, there is a need to develop machine learning models capable of determining whether allocating a specific donor organ to a particular patient would result in the patient's survival. Such models should be based on the experiences of various clinics or regions. Second, but equally important, machine learning models should be applied to analyze the factors influencing surgical outcomes. We see good potential for applying decision trees and other related models.
